# Recent advances in pain management based on nanoparticle technologies

**DOI:** 10.1186/s12951-022-01473-y

**Published:** 2022-06-18

**Authors:** Soraya Babaie, Arezou Taghvimi, Joo-Hyun Hong, Hamed Hamishehkar, Seongpil An, Ki Hyun Kim

**Affiliations:** 1 Physical Medicine and Rehabilitation Research Center and Student Research Committee, Tabriz University of Medical Sciences, Tabriz, Iran; 2 Biotechnology Research Center, Tabriz University of Medical Sciences, Tabriz, Iran; 3 School of Pharmacy, Sungkyunkwan University (SKKU), Suwon, 16419 Republic of Korea; 4 Drug Applied Research Center, Tabriz University of Medical Sciences, Tabriz, Iran; 5 SKKU Advanced Institute of Nanotechnology (SAINT) and Department of Nano Engineering, Sungkyunkwan University (SKKU), Suwon, 16419 Republic of Korea

**Keywords:** Nanomedicine, Nanoparticle, Analgesia, Pain, Nanotechnology

## Abstract

**Background:**

Pain is a vital sense that indicates the risk of injury at a particular body part. Successful control of pain is the principal aspect in medical treatment. In recent years, the advances of nanotechnology in pain management have been remarkable. In this review, we focus on literature and published data that reveal various applications of nanotechnology in acute and chronic pain management.

**Methods:**

The presented content is based on information collected through pain management publications (227 articles up to April 2021) provided by Web of Science, PubMed, Scopus and Google Scholar services.

**Results:**

A comprehensive study of the articles revealed that nanotechnology-based drug delivery has provided acceptable results in pain control, limiting the side effects and increasing the efficacy of analgesic drugs. Besides the ability of nanotechnology to deliver drugs, sophisticated nanosystems have been designed to enhance imaging and diagnostics, which help in rapid diagnosis of diseases and have a significant impact on controlling pain. Furthermore, with the development of various tools, nanotechnology can accurately measure pain and use these measurements to display the efficiency of different interventions.

**Conclusions:**

Nanotechnology has started a new era in the pain management and many promising results have been achieved in this regard. Nevertheless, there is still no substantial and adequate act of nanotechnology in this field. Therefore, efforts should be directed to broad investigations.

**Graphical Abstract:**

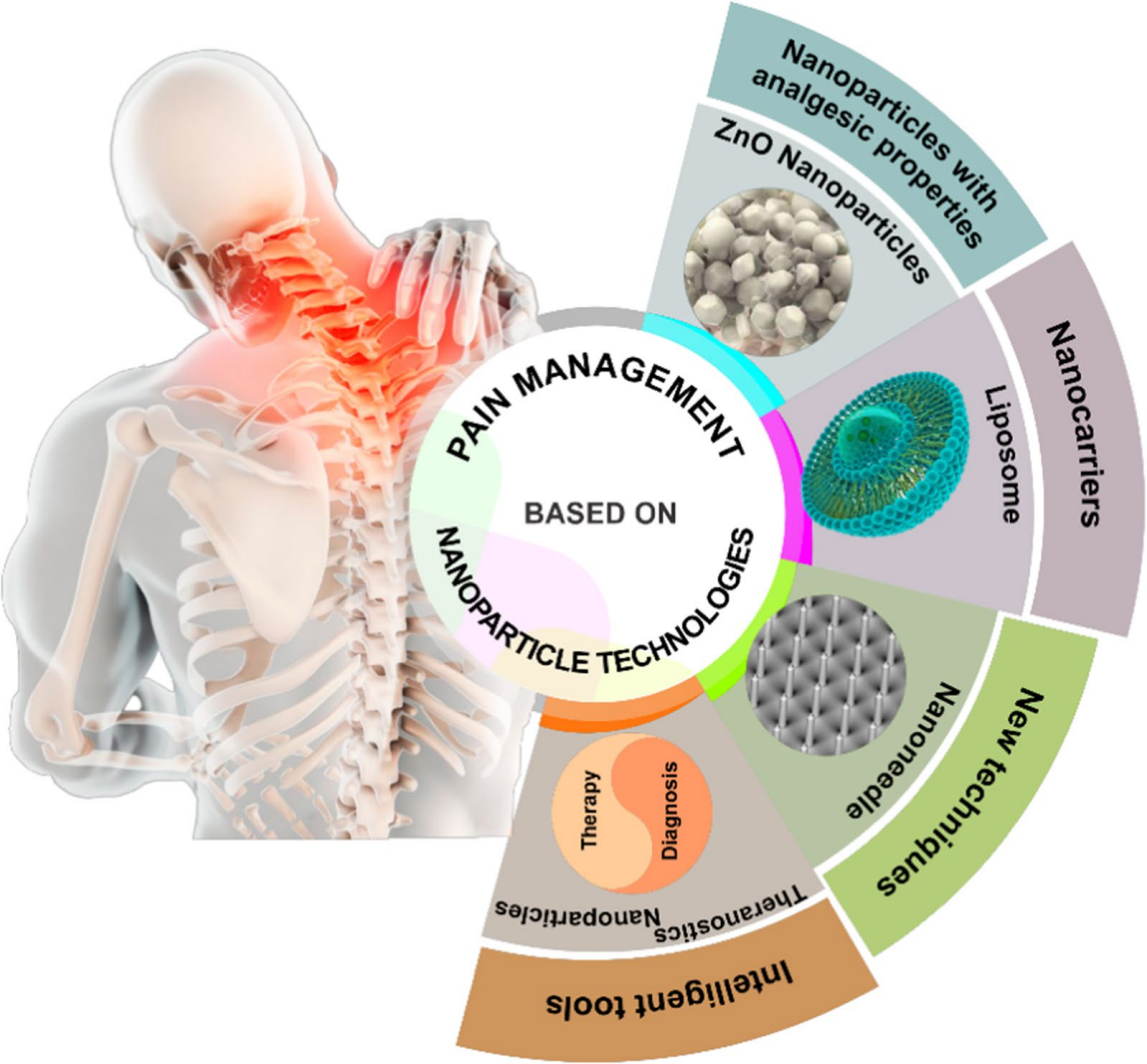

## Keypoints

- Zinc oxide (ZnO) and magnesium oxide (MgO) nanoparticles have themselves the intrinsic analgesic properties.
- The emergence of theranostic nanomedicine provides an ideal method for accurate pain management.
- Nanoneedles are new opportunities in the pain management.
- Many nanoliposomal analgesic formulations have been industrialized and are currently on the market.

## Introduction

The defensive mechanism of human body toward stimulants is demonstrated as pain, which is a common sign of diseases. Stimulants create pain by irritating free nerve endings in nociceptors (*i.e.*, pain receptors) [1], wherein histamine, leukotrienes, substance P, acetylcholine, bradykinin, and prostaglandins are some of the chemical mediators that are responsible for the pain. On the other hand, vasodilatation, vasoconstriction, and changed capillary permeability result from these chemical mediators at the injury site [2].

Pain can be described as acute or chronic in terms of clinical aspect [3]. Insufficient pain management has considerable adverse effects, for example, inadequate control of acute pain can lead to serious medical complications, such as deep venous thrombosis and pneumonia, and progresses to chronic pain. In addition, poorly treated chronic pain can result in declined daily activities of the patient, decreasing the quality of life [4, 5]. Pain based on pathophysiology is generally classified as nociceptive or neuropathic pain. Nociceptors are receptors particularly sensitive to stimulants or tissue trauma that can create tissue damage if the exposure or incidence is prolonged. On the other hand, pain originating in the periphery is not always a nociceptive pain. Neuropathic pain can be produced by abnormal signal processing in the central or peripheral nervous system [6–8].

As with types of pain, several methods exist for the treatment of pain, and generally, they can be broadly categorized into pharmacological and non-pharmacological approaches. Pharmacological strategies include the use of analgesics and analgesic adjuvants (*i.e.*, local anesthetics, nonsteroidal anti-inflammatory drugs, antiepileptic drugs, α2-adrenoceptor agonists, antidepressants, and opiates) to treat and relieve pain [9]. Whereas, non-pharmacological strategies refer to physical (*i.e.*, acupuncture, hot and cold treatment, transcutaneous electrical nerve stimulation (TENS), and progressive muscle relaxation) and psychological (mindfulness-based stress reduction and cognitive behavioral therapy) interventions that can decrease fear, anxiety and pain intensity, and also support patients to play an active role in managing their symptoms [10].

Although various clinical studies have hitherto provided progressive results in the suppression of pain for the last decade [11], since the treatment of pain is very complex, the choice of effective and safe treatment for satisfactory pain management is still very difficult even for healthcare professionals. On the other hand, current therapeutics are not adequate to relieve some types of pain, and in many cases these treatments offer debilitating side effects. Furthermore, limitations in the current detection technologies make a major defect in accurate diagnosis of pain initiators, especially in the psychiatric patients they may have an augmented perception of pain or in older adults numerous pain producers commonly coexist [12]. According to recent investigations specific biomarkers have revealed in the pain conditions [13]. The specificity and sensitivity in the detection of these biomarkers will provide ultimately accuracy in the pain management. Nevertheless, restrictions in the pain assessment methods and consequently unsatisfactory treatments need for new skills and alternative approaches. Meanwhile, pain management with employing nanotechnology has recently emerged as a new approach to improve the quality of treatment. In particular, the use of nanoparticles that have small dimension and large surface area, resulting in various special characteristics, has been allowing one to confer new strategies for the complex treatments that could not otherwise be treated [14]. This has indeed facilitated the application of nanoparticles to various fields of pain management, such as providing new possibilities towards the detection, prevention and treatment of pain. Advances in the introduction of nanoparticles with intrinsic analgesic properties that can modulate pain perception with high satisfaction have provided a new perspective in the pain management [15]. Development of engineered multifunctional nanoparticles such as lipid-based nanoparticles represent a promising strategy to improve the low therapeutic index of conventional analgesic drugs and are already used in clinical practice [16]. Polymeric nanoparticles such as surface modified PLGA nanoparticles and another influential carriers such as silica, gold and magnetic nanoparticles extremely have been investigated to pain modulation [17–20]. Over the last few years, various nanomaterials have hiterto provided the accurate detection of biomarkers and molecular sources of pain to reduce the dose of analgesics and increase their long-term efficiency and safety. More importantly, the emergence of theranostic nanoparticles, which is a combination of diagnosis and treatment, has provided the basis for accurate pain management. Despite the considerable results in preclinical studies, limited clinical trials have been reported for nanomaterials in the control of pain, and only a few nanomedicines could obtain FDA approval [21]. Among the various nanoparticles, liposomes have been the most clinically studied for the delivery of analgesic drugs, and the results have shown the strong potential of these nanoparticles in the safe management of pain [22]. It is essential that the studies should focus on the use of products in clinical applications, while also paying special attention to their safety and effectiveness.An appropriately synthesized nanoparticle through accurate surface modification, particle size reduction, and acceptable encapsulation facilitates the development of an effective formulation for pain control. Although, there are several review articles about the role of nanomaterials in the pain management, this review article especially focuses on the introduction of novel nanoparticles with intrinsic analgesic properties and new nanosystems for targeted delivery of analgesic drugs. In addition, this review discusses the discovery of new nanotechniques and application of intelligent tools facilitating accurate control of pain, and also highlights current clinical studies and FDA approved products in this area.

### Nanoparticles having intrinsic analgesic properties

New advances in nanomedicine have led to the emergence of various nanoparticles with unique properties. This has indeed enabled the expansion of the application of nanoparticles to various fields of medicine such as providing new possibilities towards the detection, prevention and treatment of diseases such as cancer, diabetes, cardiovascular disease, Alzheimer, Parkinson and other life-threatening disorders [23–27]. However, to date, studies focusing on the analgesic effect of nanoparticles are limited. In the following section, examples of nanoparticles with analgesic property are described.

### Zinc oxide (ZnO) nanoparticles

**Fig. 1:**
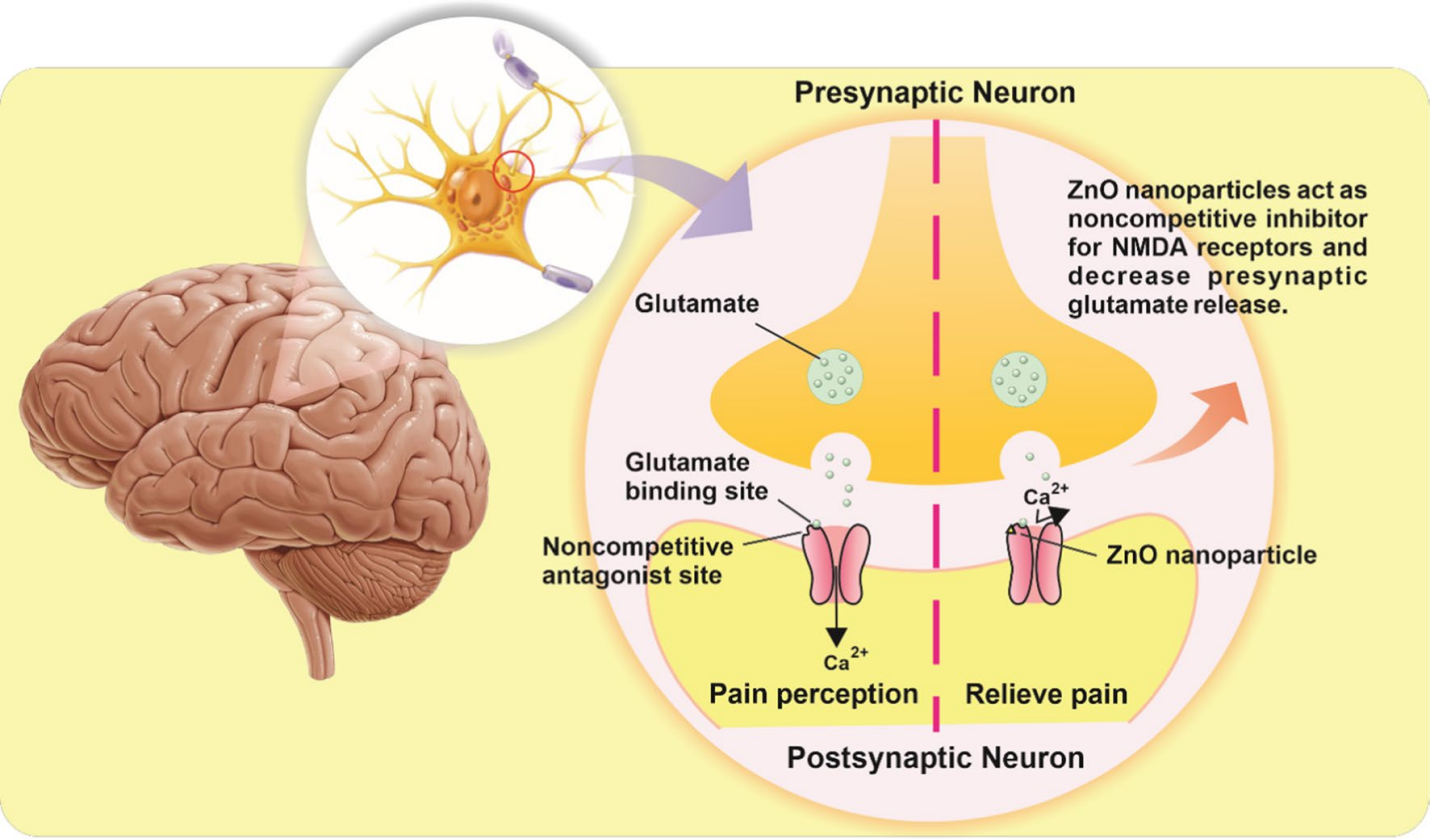
Mechanism of the action of ZnO nanoparticles with analgesic property. ZnO nanoparticles reduce glutamate release. They are noncompetitive inhibitors of NMDA glutamate receptors; thus, they decrease the capability of glutamate effect in these receptors

Various forms of ZnO, including nanoparticles, nanowires, etc., have exhibited great applicability in various fields, such as sensors, cosmetics, secondary batteries, optoelectrical devices, solar cells, and drug delivery [28–36]. In particular, ZnO nanoparticles have recently attracted great attention in biomedical fields because of its zero-dimensional structure and electrochemical properties [e.g., nontoxicity, wide band gap (~ 3.3 eV), and high electron mobility (~ 2000 cm^2^/V s at 80 K)]. Zinc is one of the essential and trace elements that can affect on the pain sensation. [37, 38]. Indeed, recent studies have explored a relationship between zinc deficiency and antinociceptive effects, in which they have particularly focused on interactions between zinc availability and opioidergic system activity. For example, it is demonstrated that zinc deficiency in diet could lead to lower antinociceptive effect of morphine in mice [39]. In addition, one study reported that ZnO nanoparticles could reduce the pain through the activation of the opioidergic system [15]. Zinc, which is a noncompetitive inhibitor of N-methyl-d-aspartate (NMDA) glutamate receptors, generally acts in postsynaptic space on most of receptors and channels; thus, it can decrease the capability of glutamate effect in this receptor. Note that NMDA is recognized as one of the important receptors in the pain management process. That is, the use of ZnO nanoparticles could block the NMDA receptors and induce the analgesic effect (Fig. 1) [40], wherein an increase in the levels of γ-aminobutyric acid (GABA) as an inhibitory neurotransmitter also reduced glutamate release, and may also induce the analgesic effect of ZnO nanoparticles [41].

On other hand, since the form of nanoparticles has more chemical reactive sites than the materials in bulk form because of the high surface-to-volume ratio, it is known that the chemical reactivity of ZnO nanoparticles is stronger than that of the conventional ZnO. Indeed, the analgesic effect by the ZnO nanoparticles could be achieved in doses lower than those of the conventional ZnO in bulk form. In addition, the extremely-small scale of ZnO nanoparticles allowed them to easily penetrate through body barriers and interact well with cell components, which could also result in the significant promotion of reactivity effects compared to the conventional ZnO in bulk form. For instance, although both ZnO bulk form and ZnO nanoparticles demonstrated analgesic effect on acute pain, the use of ZnO nanoparticles exhibited a higher analgesic effect than the ZnO in bulk form at a hot plate test with Wistar rats [15].

### Magnesium oxide (MgO) nanoparticles

**Fig. 2:**
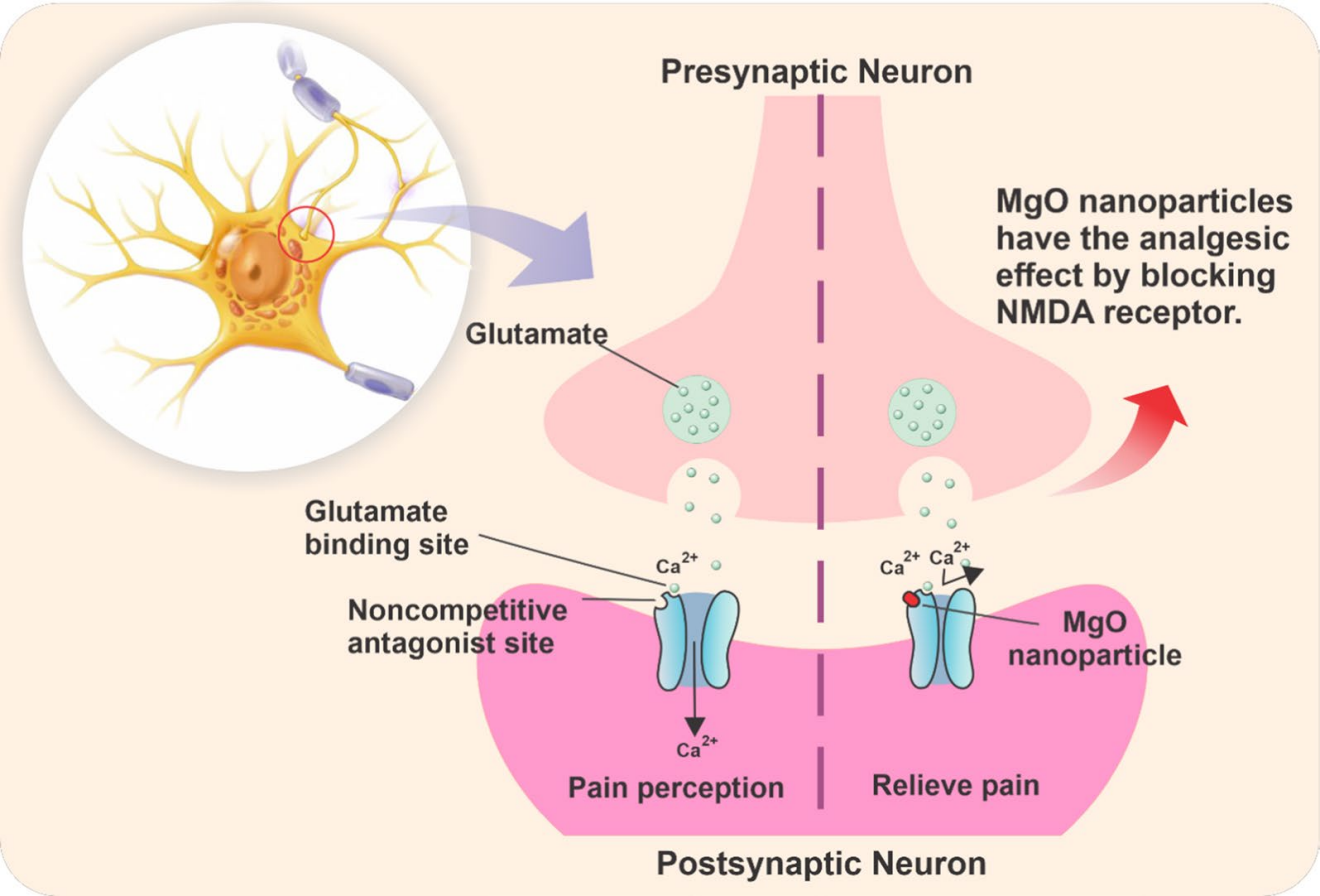
Mechanism of action of the MgO nanoparticles with analgesic property. MgO nanoparticles block NMDA receptors, inducing the analgesic effect

Magnesium is the fourth most important cation in human body, and the second most common intracellular cation [42]. Earlier, magnesium sulfate was suggested as a general anesthetic. The antinociceptive effect by using magnesium in the animal and human models of pain is related to the blocking of NMDA receptor. Magnesium without any direct analgesic effects, prevents calcium ions from entering cells by blocking NMDA receptors, resulting in an antinociceptive effect [43] (Fig. 2). Hence, the use of magnesium could lead to decrease the requirement of relaxant drugs and intraoperative anesthetics and the amount of morphine required for postoperative pain treatment. Such effects of magnesium were yielded not only in general anesthesia, but also in local anesthesia [44]. Moreover, another antinociception mechanism derived from the use of magnesium is associated with calcium-antagonistic effects at various voltage-gated channels [45].

The analgesic effects of MgO nanoparticles on anesthesia were demonstrated by Jahangiri et al*.*, wherein their experimental testes confirmed strong analgesic effects of MgO nanoparticles in comparison to conventional MgO. According to this group, this subject may be related to the high permeability effect of MgO nanoparticles, especially in the central nervous system [46]. Another study was designed to estimate the effect of MgO nanoparticles and conventional MgO on ketamine-induced anesthesia in rabbits, the results showed that duration and quality of anesthesia induced by ketamine were similar in MgO nanoparticles and conventional MgO [47].

### Manganese dioxide (MnO_2_) nanoparticles

**Fig. 3:**
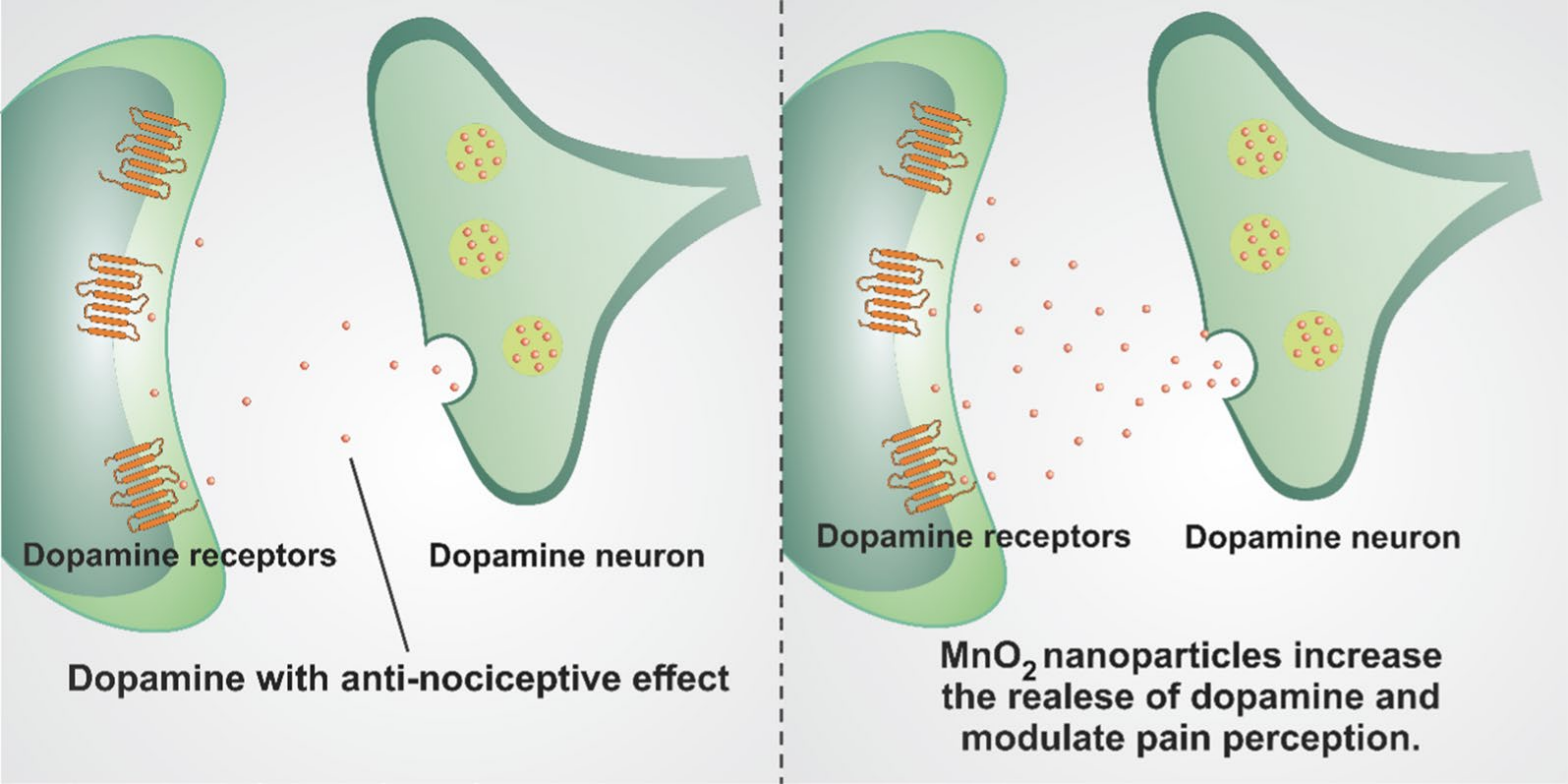
Mechanism of the action of MnO_2_ nanoparticles with analgesic property. MnO_2_ nanoparticles affect the dopaminergic system and modulate analgesia

Various clinical and animal studies demonstrated the antinociceptive effects of dopamine [48–50]. For example, loss of dopaminergic neurons in patients with Parkinson’s disease caused an increase in pain perception [51]. MnO_2_ can influence the dopaminergic system, modulating analgesia and pain perception [52] (Fig. 3). wherein reduced levels of dopamine probably cause the painful signs and irregularities in dopaminergic neurotransmission have been obviously revealed in painful clinical disorders, including burning mouth syndrome and restless legs syndrome [53].

Pain thresholds of rats were assessed using the tail immersion method to study the effect of MnO_2_ micro- and nanoparticles after a single subcutaneous injection. The result indicated that both nano- and micro-sized MnO_2_ particles could pass through blood barriers; however, the change in the particle size influenced the Mn clearance and distribution from the central nervous system (CNS). During 8 weeks of the microparticle administration, a steady and irreversible increase in the pain thresholds was observed in the rats while the MnO_2_ nanoparticles presented a biphasic response as follows: primary hyperalgesia for the first 4 weeks and late analgesia during the next 4 weeks. Note that a decrease in descending pain modulation tract activity promotes hyperalgesia. In the first 4 weeks, the observed hyperalgesia was due to the effect on the central descending mechanisms that stimulated the hyperalgesia and mask the analgesic effect of these nanoparticles, which was obtained by acting on the dopaminergic system. In the last phase of the experiment, the long-term analgesic effect of MnO_2_ nanoparticles was observed because of the effect on the dopaminergic system [52].

### Magnetite (Fe_3_O_4_) nanoparticles

Magnetite (Fe_3_O_4_) is one of the most explored magnetic nanoparticles for biomedical applications. The unique properties of these nanoparticles have expanded their application in various fields of medicine. The most unique feature of these particlesis their ability to be conducted by an external magnetic field; therefore, more examinations have been motivated based on this property, such as development of contrast agents for magnetic resonance imaging (MRI), cell tracking, bioseparation and targeted drug and gene delivery [54–56].

**Fig. 4:**
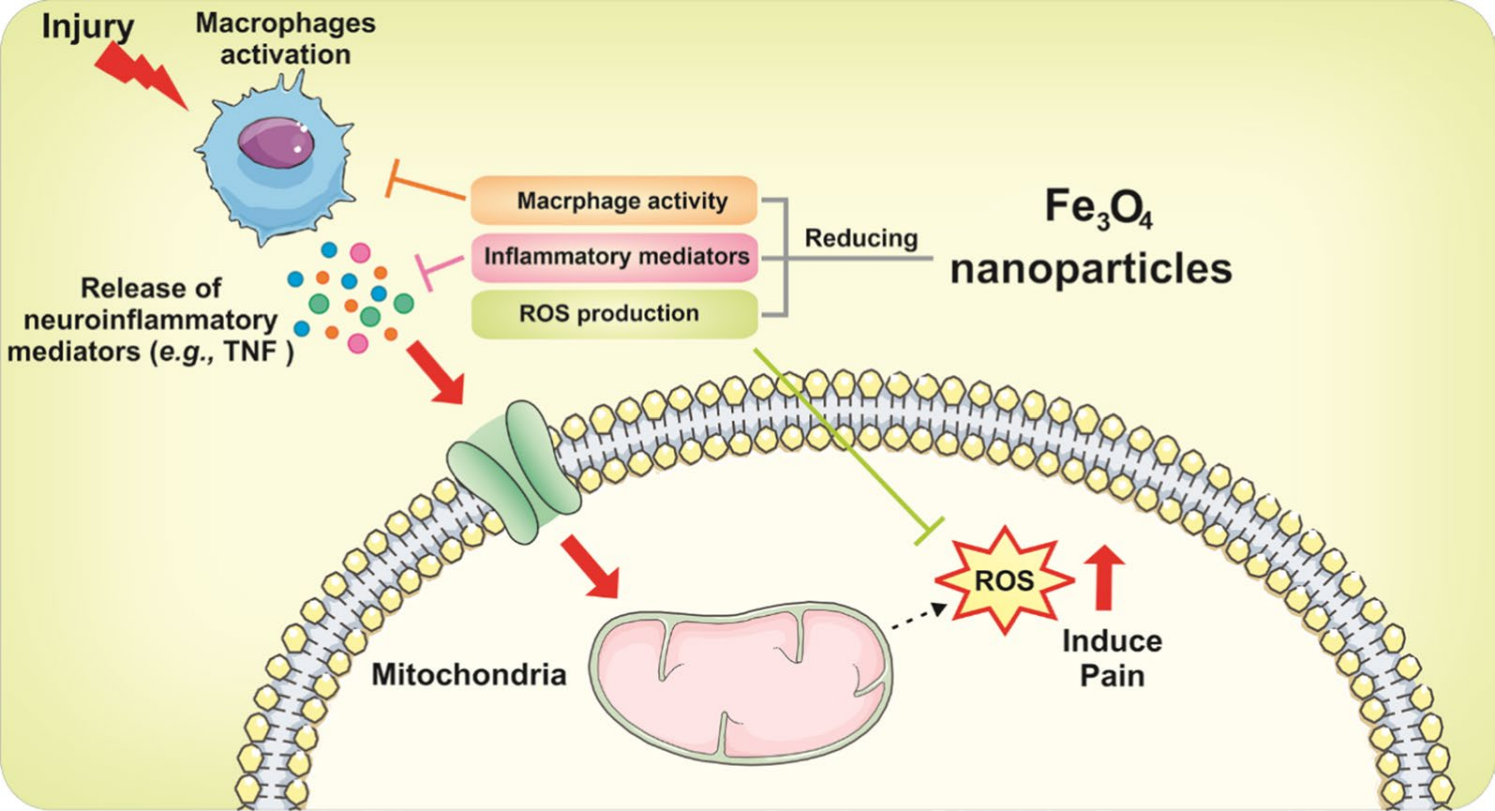
Mechanism of action of the Fe_3_O_4_ nanoparticles with analgesic property. Fe_3_O_4_ nanoparticles induce analgesia by the suppression effect on macrophage activity and a decrease in ROS production, inflammatory cells, and proinflammatory markers

However, its use as a drug carrier has been restricted to the CNS disorders. Accordingly, few studies have hitherto reported the analgesic effect of Fe_3_O_4_ nanoparticles. Wu et al*.* developed inflammatory pain models in CD1 mice by injecting complete Freund’s adjuvant [57]. The result indicated that the dose-related analgesia was obtained by local administration of the ultrasmall Fe_3_O_4_ nanoparticles via the suppression effect on macrophage activity, inflammatory cells, and proinflammatory markers and the significant decrease in reactive oxygen species (ROS) production in the injured paw (Fig. 4).

In another study, they reported excellent analgesia production by the polarity modulation of magnetic field to guide the intrathecal delivery of ketorolac-Fe_3_O_4_ nanoparticle conjugate [58]. The ketorolac was covalently conjugated onto the Fe_3_O_4_ nanoparticles, which could prevent the early release of ketorolac before reaching the target site. In this study, it was suggested that ketorolac–magnetic nanoparticles conjugate may break down to free ketorolac and magnetic nanoparticles in cerebrospinal fluid and act synergistically to produce an analgesic effect [59]. In other words, the Fe_3_O_4_ nanoparticles acted as nanocarriers and pharmaceutical units and had a critical role in increasing local drug accumulation via magnetic field modulation.

On the other hand, the Fe_3_O_4_ nanoparticles were also used as nanocarriers for ankle analgesia, in which the corresponding analgesic effect thereof was investigated [60]. In order to replace the existing nerve block methods, Mantha et al*.* conducted tests on the ankle block in the rat with intravenous injection of the Fe_3_O_4_ nanoparticles incorporated with ropivacaine (Fe_3_O_4_/Ropiv) in addition to the use of a magnet at the ankle site. Intravenous injection of the Fe_3_O_4_/Ropiv with magnetic force at the ankle site considerably could augment paw withdrawal latencies. Note that the intravenous injection with the Fe_3_O_4_ nanoparticles only had no major influence on paw withdrawal latency.

### Nanoparticles designed for analgesic drugs and nanocarriers

**Fig. 5:**
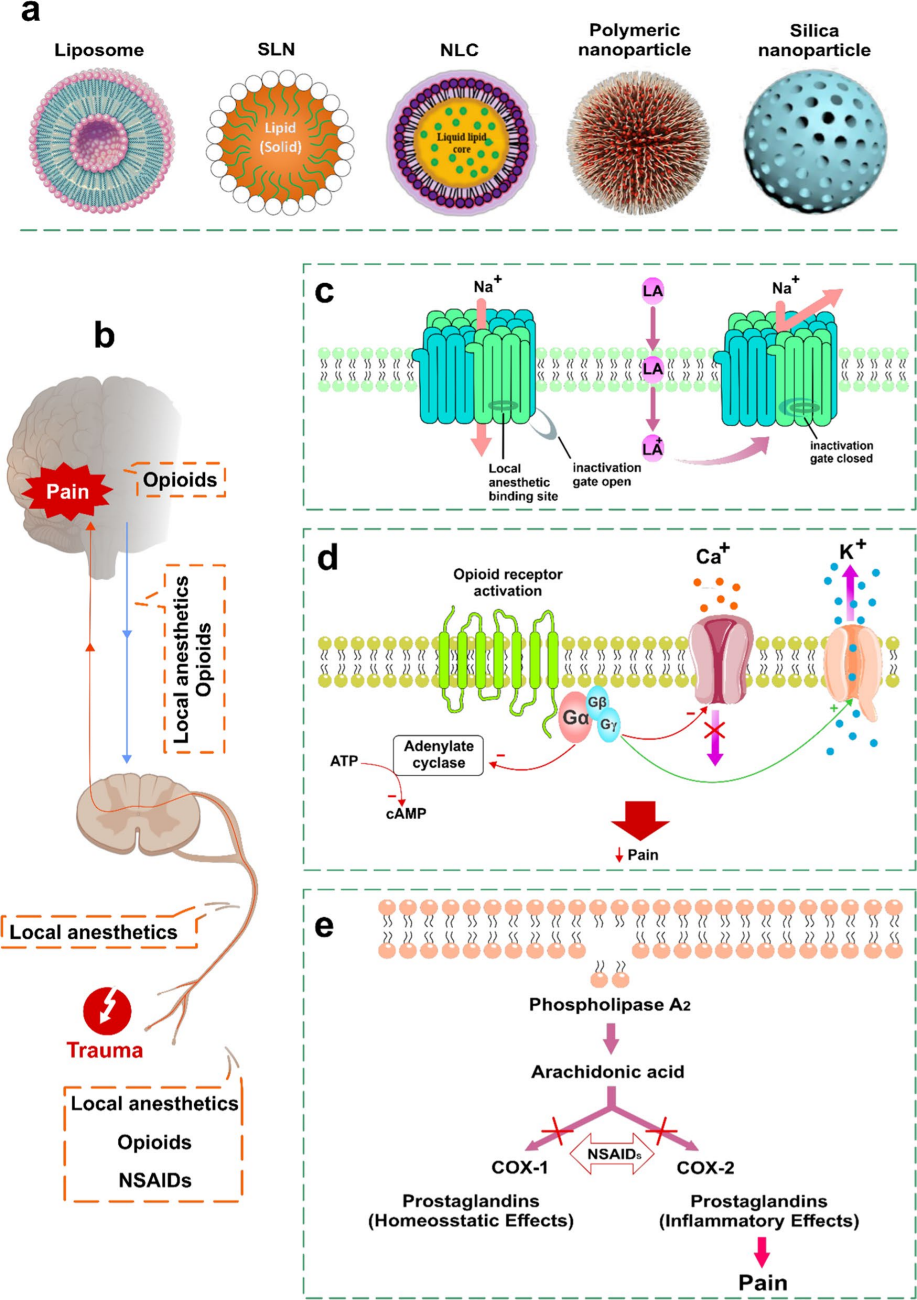
Illustrations of the nanocarriers designed for (**a**) analgesic drugs, **b** therapeutic modulation of the pain pathway, and the working mechanism of (**c**) local anaesthetics, (**d**) opioids, and (**e**) NSAIDs for pain management

Pharmacological therapy using analgesic drugs is one of the main methods for pain treatment. According to one of the analgesics classifying systems, analgesics can be classified as opioid analgesics (*i.e.*, morphine-like agonists), adjuvant analgesics (*i.e.*, local anesthetics), and nonopioid analgesics [*i.e.*, nonsteroidal anti-inflammatory drugs (NSAIDs)]. Targeted delivery and sustained release of analgesic drugs remains a critical problem for effective control of pain. The use of nanocarriers has been informed to improve effective delivery of these agents to target sites while reducing side effects. Accordingly, current advances in the presentation of novel nanocarriers such as lipid based nanocarries and polymeric nanoparticles will definitely be effective in the quality of pain management (Fig. 5a) [61, 62].

### Nanoparticles designed for opioids

Consumption of opioids for chronic pain management has dramatically increased in recent years. However, there are some concerns about its analgesic efficacy because of tolerance to opioid therapy. For example, it is known that despite increasing doses the efficacy of opioids decreases during the course of treatment [63]. Opioids exert their actions by interacting with specific receptors present on the central and peripheral nervous system. Activation of opioid receptor reduces the formation of intracellular cyclic adenosine monophosphate (cAMP) and opens K^+^ channels or suppresses Ca^2+^ channels. All of these events lead to neural hyperpolarization and reduced intracellular Ca^2+^ availability, thereby reducing the release of CNS neurotransmitters and inducing analgesia (Fig. 5d) [64]. Many opioids, such as morphine and hydromorphone, have a very short half-life. For the opioid to be effective it is necessary to increase the frequency of administration, however, that can increase the incidence of adverse effects and inconsistent drug levels [65, 66]. For these reasons, the proper modulation of drug adsorption is required after administration. Accordingly, recent pharmaceutical formulation studies have focused on the development of drug delivery systems that have the potential for sustaining or delaying drug release after administration [67].

One of the well-established nanomaterials, liposome is a spherical vesicle containing phospholipid bilayers, which generally have the size of tens of nm to thousands of nm. These vesicles can trap lipophilic or hydrophilic drugs inside, and thus can release their contents at the target site [68]. For instance, *Depodur* approved by the food and drug administration (FDA) for postoperative pain management, which is the prolonged-release epidural morphine, could be encapsulated at the inside of multivesicular liposomes (*i.e.*, *Depofoam*). In particular, this *Depofoam* includes several encircled bilayers capable of making numerous aqueous compartments for morphine salt preservation [69, 70].

Inspired by such products, significant advances in the field of oral delivery of opioids have been also achieved using the technologies associated with liposomes. However, when native-state liposomes are orally administrated into the body, they are rapidly degraded in harsh gastrointestinal (GI) conditions. Coating the liposomes with polymers conferred stability in the GI tract. Surface modification of liposomes by polyethylene glycol (PEG) and Eudragit^®^ S 100 copolymer have improved the oral bioavailability of tramadol and endomorphin-1, respectively, by prolonging the blood circulation of the liposomes [71, 72].

Peptide-coated liposomes are another example of strategies to enhance analgesic effects of opioids following intranasal administration. The use of the upper nasal cavity for delivery of opioids can directly distribute the opioids into the brain. Arg–Gly–Asp (RGD) peptide sequence is an integrin-targeting ligand while the surface modification of liposomes by the RGD peptide could increase the residence time and penetration ability through epithelial layers. In this regard, Hoekman et al*.* reported aerosol liposomal formulation coated with the RGD peptide for the nasal delivery of fentanyl, where they could demonstrate improvement in analgesic effect in comparison with the free drug formulation [73]. Similarly, improvement in the intrathecal and transdermal deliveries of alfentanil, butorphanol and buprenorphine could be also achieved using the liposomal formulation in other studies [74–76].

On the other hand, since 1990 so-called solid lipid nanoparticles (SLNs) and nanostructured lipid carriers (NLCs) have been often reported as the alternative carrier systems for emulsions, polymeric nanoparticles, and liposomes [77]. The NLCs are regarded as the next generation of SLNs because of the enhanced loading capacity and stability, and decreased drug expulsion during storage [78]. Note that the opioids are also the drugs of choice for pain control in burn or skin graft patients. However, managing severe pain in burn and skin graft patients is still challenging because common formulations have insufficient pain control because of inadequate concentrations and degradation of drugs at the target site. To enable topical opioid application to these wounds, Küchler et al*.* developed morphine-loaded SLNs for the effective reduction in pain and acceleration of wound healing [79]. In another successful study, in vivo assessments indicated an improvement in analgesic effect of fentanyl ﻿citrate-loaded NLC in comparison to the commercial type [80].

### Nanoparticles designed for local anesthetics

Local anesthetic is a class of anesthetic drugs capable of numbing a specific part of body by blocking Na^+^ channels and suppressing action potentials. Local anesthetics pass through the membrane in unionized lipophilic form and ionize again in the axoplasm, where they attach to their receptors and stabilize the channel in the inactive state, thus reducing channel opening and inducing analgesia (Fig. 5c) [81]. Since a patient after the local anesthetic can be awake or sedated during local anesthesia depending on the option, this process is often used for minor outpatient procedures [82]. Meanwhile, many researchers have hitherto studied actively to increase the duration of these local anesthetics. In order to control the drug release of local anesthetics, researchers have focused on the combination of local anesthetics and carriers, in particular, they have employed technologies of nanocarriers, such as liposomes, lipid nanoparticles, and polymeric nanoparticles. Table 1 presents a summary of these studies that revealed the efficiency of various nanoformulations in local anesthesia.

**Table 1:** Experimental studies with local anesthetics encapsulated into various nanoparticles

Local anesthetic drug	Nanoparticles	Subjects (administration route)	Antinociceptive test	Results	Ref
Benzocaine	PLGA, PLA, PCL	Mice (injection into the popliteal space)	PWTP	Pharmacological assessment indicated that encapsulation of benzocaine in polymeric nanocapsules prolonged its anesthetic effect compared with free benzocaine	[83]
Lidocaine/prilocaine	Polymeric nanocapsules	Rat (oral mucosa)	Tail-flick	The formulation provided effective and longer-lasting superficial anesthesia at the intraoral mucosa during medical and dental procedures	[84]
Bupivacaine	Alginate/chitosan (ALG–CHIT)-Alginate/bis (2-ethylhexyl) sulfosuccinate (ALG–AOT)	Mice (popliteal space)	PWTP	Comparison among formulations displayed that 0.125% bupivacaine-loaded ALG–CHIT and bupivacaine-loaded ALG–AOT improved the analgesia 1.5- and 2.16-fold, respectively, compared with plain bupivacaine	[85]
Ropivacaine	Liposome	Human (oral mucosa)	Pinprick test	Extensive soft tissue anesthesia was achieved using liposome containing ropivacaine and EMLA group, when compared with ropivacaine and benzocaine gels (*P* < 0.0001)	[86]
Tetracaine	Liposome	Human (skin)	McNemars test	The formulation produced better superficial local anesthesia than EMLA	[87]
Bupivacaine	Alginate/chitosan	Rabbit-rat (intraoral or intrathecal injections)	Observation of the aversive response to the rat upper lip pinching	The analgesic properties of bupivacaine increased 1.4-fold (*P* < 0.001) with bupivacaine 0.5%-loaded alginate–chitosan nanoparticles after infraorbital nerve blockade	[88]
Tetrodotoxin	Hollow silica nanoparticles	Rat (sciatic nerve injection)	Neuro-behavioral testing	The sustained release of tetrodotoxin encapsulated in hollow silica nanoparticles with diameter of 28 nm prolonged duration of action and at the same time reduced systemic toxicity	[89]
Tetrodotoxin	Poly(triol dicarboxylic acid)-co-poly(ethylene glycol)	Rat (sciatic nerve injection)	Neuro-behavioral testing	Tetrodotoxin released from polymers created nerve block duration, from several hours to 3 days, with insignificant local or systemic toxicity	[90]

*PWTP* paw withdrawal threshold to pressure, *PLGA* poly(lactic-co-glycolic acid), *PLA* polylactic acid, *PCL* polycaprolactone, *EMLA* eutectic mixture of local anesthetics

Liposome is one of the primary drug delivery systems that have been used for decades to deliver local anesthetic drugs for skin analgesia or management of postsurgical pain. The first local anesthetic drug used with a nanocarrier was suspension of bupivacaine-containing liposomes, introduced by Boogaerts et al. Although epidural injection of the liposomal formulation demonstrated that this formulation was not appropriate for anesthesia during surgery, further studies could reuse this approach to control postoperative pain [91]. The success of liposomes in the delivery of local anesthetic drugs has been also able to lead to the development of products, such as *EXPAREL* (bupivacaine liposome injectable suspension). The *EXPAREL* is formulated to produce postsurgical local analgesia, *i.e.*, interscalene brachial plexus nerve block after single-dose infiltration. Intraoperative administration of the *EXPAREL* through wound infiltration could significantly reduce pain over 72 h compared with a placebo [92].

Anesthesia is one of the most commonly used methods for pain relief, which can be generally conducted by the insertion of a needle and the injection of a local anesthetic agent. Elimination of the needle for local anesthesia especially using nanoparticle technologies would be a great step in most invasive procedures on skin and dental treatments. In particular, the delivery of local anesthetics through the skin has many benefits, such as greater patient compliance, extended sustained drug release, rarer systemic adverse reactions, and easier application [93]. In general, when local anesthetics-loaded nanocarriers are applied to the skin, they can penetrate skin layers by bypassing the main skin barrier, *i.e.*, stratum corneum. Moreover, bioadhesives containing local analgesic agents can effectively relieve pain produced by the needle stick injections, for example, in dental procedures. In this regard, to provide topical, gingival, and mucosal anesthesia, the lidocaine transmucosal delivery system (*DentiPatch*) was developed. Wherein, applying this mucoadhesive patch minimize the pain sensation prior to dental procedures such as local anesthetic injections. Therefore, use of an effective topical anaesthetic agent with longer duration of action will eventually lead to more comfortable for both patients and clinicians [93, 94]. Many of local anaesthetics mentioned above are non-specific blockers of the sodium channel. The toxins such as tetrodotoxin and saxitoxins, resulting from various sources are very potent and specific blockers of the voltage gated sodium channels. These toxins bind to the outer pore and block site 1 on the voltage-gated sodium channel. The charge and hydrophilicity cause the relatively weak penetration through various barriers to their site of action and limit the efficiency of these local anesthetics [95]. In an attempt to overcome these limitations and increase nerve penetration, hollow silica nanoparticles containing tetrodotoxin were evaluated. These nanoparticles are popular candidates because the hollow structure and negative charge of silica can facilitate the encapsulation of tetrodotoxin. In this study, it was found that the ability of silica nanoparticles to penetration was highly size-dependent and sustained release of tetrodotoxin prolonged duration of action and at the same time reduced systemic toxicity [89].

On the other hand, researchers must have attention to improve drug loading to overcome the factors that reduce the potency of local anesthetics. To achieve this, each anesthetic drug should be compatible with physicochemical features of the nanocarriers, such as molecular shape, lipid solubility, charge, and polarity. Table 1 represents a comprehensive list of local anesthetic drugs encapsulated into different nanoparticles along with details about administration routes and antinociceptive tests for pain assessment of these formulations.

### Nanoparticles designed for NSAIDs

NSAIDs are the class of drugs widely prescribed to control pain, which are known as very effective against inflammation and pain. The main action mechanism of these drugs is inhibition of cyclooxygenase (COX) enzyme, which metabolizes arachidonic acid to prostaglandins, causing pain and inflammation (Fig. 5e) [96]. However, they have shown many side effects, including GI bleeding, nephrotoxicity, and cardiovascular side effects [97]. These side effects of NSAIDs have been major limitation that hampers their practical uses in pain management and inflammatory conditions. Thus, recent studies have focused on improving efficacy and reducing side effects of NSAIDs [97], in particular, several nanotechnologies, such as liposomes, SLNs, dendrimers, micellar-, silica-, and polymeric nanoparticles, with various compounds have been actively studied to improve the effectiveness of NSAIDs. Table 2 presents a summary of studies that examined the efficacy of various nanoparticles having NSAIDs. These nanoparticles were designed for targeted drug delivery, controlled drug release, increased drug efficacy, and prevention of destruction of drugs [98, 99].

**Table 2:** Experimental studies with NSAIDs encapsulated into various nanoparticles

NSAID	Nanoparticles	Route of administration	Results	Ref
Piroxicam	Ethyl cellulose	Oral/rat	Piroxicam-loaded ethyl cellulose nanoparticles significantly decreased (approximately 66%) gastric ulceration in rats in comparison to piroxicam suspension	[100]
Naproxen	PLGA	In-vitro analysis	Formulation of naproxen–PLGA nanoparticles could improve the physicochemical characteristics of the drug	[101]
Meloxicam	Eudragit EPO	Oral/rat	The EPO nanoparticle significantly increased anti-inflammatory activity of meloxicam (for longer duration; 6 h) in comparison to meloxicam suspension	[102]
Ibuprofen sodium	Gelatin	Oral/rat	Nanogelatin improved plasma half-life of ibuprofen sodium, thereby aiding reduction in the frequency of administration	[103]
Flurbiprofen	Cyclodextrin (CD) complexation and liposomes	Intravenous/rat	This delivery system significantly enhanced the bioavailability of flurbiprofen	[104]
Indomethacin	Chitosan-coated liposomes	Oral/rat	Results showed that retention in the upper part of the GI tract was better for submicronized chitosan-coated liposomes in comparison with submicron-sized liposomes, at 1, 2, and 4 h after administration, and the nanosystem was significantly better retained in the small intestine at 4 h	[105]
Nimesulide	Lipid	Intraplantar injection/rat	The nanoparticles offered significant pharmacological effects in comparison with free drug administration	[106]
Naproxen	Nanostructured lipid carrier (NLC)	Temporomandibular joint/rat	Proinflammatory cytokines (IL-1β and TNF-α) and leukocytes migration significantly decreased for more than a week by sustained delivery of naproxen directly in the temporomandibular joint	[107]
Flurbiprofen	Polyvinylpyrrolidone (PVP)	Oral/rat	Nanoparticles improved the solubility of flurbiprofen by approximately 130,000-fold. This formulation improved bioavailability of poorly water-soluble flurbiprofen	[108]
Indomethacin	Poly(2-hydroxyethyl methacrylate-co-3,9-divinyl-2,4,8,10-tetraoxaspiro (5.5) undecane, polymeric nanoparticle	Oral/mice	In vitro and in vivo results indicated the potential of this polymer as a matrix for bioactive produces	[109]

*EPO* pH sensitive cationic polymer consisting of 1:2:1 ratio of methyl methacrylate; *N_N-*dimethylaminoethyl methacrylate, and butyl methacrylate monomers; *GI* Gastro intestinal; *PLGA* poly(lactic-co-glycolic acid); *IL-1β* interleukin‐1β; *TNF-α* Tumor necrosis factor α

Lipid nanoparticles have been studied to reduce side effects and improve the solubility of NSAIDs. For example, SLNs loaded with ibuprofen and a variety of matrix lipids, including stearic acid, tripalmitin, and trilaurin, have been demonstrated to increase the dissolution rate of ibuprofen [110]. The SLNs containing trilaurin exhibited rapid ibuprofen dissolution within the first 30 min when tripalmitin was used. The dissolution rate increased, and all the ibuprofen could be released within 2 h. However, in case of stearic acid, the dissolution rate was relatively slow and ibuprofen was released within approximately 5 h. Lopes-de-Araújo et al*.* have also developed NLCs to improve pharmacokinetic and pharmacodynamic properties and reduce gastric complications of oxaprozin. The macrophages are an important element of inflammatory pathways. Furthermore the folate receptor is overexpressed in activated macrophages. Therefore in this study, conjugating folic acid on the surface of NLCs stimulated an increased macrophages uptake by a caveolae uptake mechanism. Eventually, the developed formulation provides a great potential for oral administration of oxaprozin by limiting gastric side effects [111, 112].

Polymeric nanoparticles are another drug delivery system widely used to improve the bioavailability of NSAIDs. Celecoxib that was entrapped in poly(lactide-co-glycolide) (PLGA) using didodecyldimethylammonium bromide (DMAB) or poly(vinyl alcohol) as a stabilizer could be produced by the solvent evaporation method [113]. The results demonstrated that the formulation of PLGA nanoparticles loaded with celecoxib using DMAB produced stable and highly entrapped nanoparticles to provide a potential effective dosage form for oral administration of celecoxib. In another study, positively charged polymeric nanoparticles with polycaprolactone as a biodegradable polymer and DMAB as a cationic surfactant could improve therapeutic efficacy of meloxicam with exhibiting less ulcerogenic than meloxicam suspension [114]. Polymers and polysaccharides as natural polymers appear to be successful in producing suitable carriers for NSAIDs. For instance, gelatin nanoparticles containing indomethacin provided controlled release of the drug and improved oral bioavailability of indomethacin [115].

### Nanoparticles and other nanotechniques for nociceptive pain

Nociceptors are receptors that are sensitive to tissue trauma or a stimulus that can cause injury to tissue if lasted for long. When a noxious stimulus activates a nociceptor, signals transfer mostly along with two fiber types: rapidly myelinated A-delta fibers and slowly conducting unmyelinated C-fibers. These receptors are free nerve ending distributed below the skin, joints, tendons, and body organs [116]. Nociceptive pain control originating from the skin has received a lot of attention in the field of nanomedicine. Control of pain perception before any invasive procedures in the skin could be achieved by the enhancement of analgesic permeation by nanocarriers. In addition, micro- and nanoneedles are new technologies that can significantly reduce needle anxiety of patients.

### Enhanced dermal penetration by nanoparticles

Various types of invasive procedures, such as intravenous catheter insertion and intramuscular injections, are painful operations because of the comprehensive distribution of pain receptors on the skin surface [117]. To reduce pain perception of these procedures, different anesthetic options have been presented by physicians, however, inadequate dermal penetration of anesthetic drugs has been always a great challenge. Incorporation of the anesthetic drugs into nanocarrier systems overcomes minor penetration drawbacks. The nanocarriers that have been examined for enhanced skin penetration include nanoemulsions, liposomes, SLNs, and NLCs [118, 119].

Various vesicular systems, particularly liposomal family, have been studied by numerous investigators. Ethosomes are the type of liposomal systems containing phospholipid and high concentrations of ethanol (20–45%) in their structure. Babaie et al*.* studied the ethosome containing lidocaine to investigate dermal penetration and corresponding analgesic effects [120]. In vitro studies using Franz cell system and fluorescence measurement revealed that a high amount of lidocaine was concentrated at the dermis where most nerve endings are located. Therefore, their results demonstrated that the ethosomes are effective systems for the delivery of lidocaine and suitable nanocarrier for the development of skin analgesia. On the other hand, Somagoni et al*.* combined two different nanosystems, *i.e.*, nanomicelle and nanoemulsion, that was so-called nanomiemgel, which allowed one to obtain maximum drug penetration due to its unique mixture [121]. Their studies demonstrated that absorption of nanomiemgel containing capsaicin and aceclofenac was better than either of the individual nanomicelle and nanoemulsion because of the utilization of maximum possible paths for the absorption of drugs.

Lipid–polymer hybrid nanoparticles (LPNs) are core–shell structures, thus can incorporate properties of two nanoparticles, *i.e.*, liposome and polymeric nanoparticle containing lipid shells and polymer cores. Wang et al*.* explored LPNs containing lidocaine to compare their skin penetration properties with conventional liposomes [122]. In vitro examination and in vivo analysis with tail-flick test demonstrated that the LPNs were effective drug delivery systems for local anesthetic therapy. The simultaneous delivery of two local anesthetic drugs could be also performed by using SLNs and NLCs [123]. A comparison between dermal penetration and analgesic effects revealed that stronger in vivo anesthetic effect was created by the NLC system than the SLN system, whereas the SLN systems demonstrated better ex vivo skin permeation ability than that of the NLCs. That is, it could be concluded that both nanoparticle technologies with various prominent characteristics can be effectively employed as suitable nanocarriers for topical skin analgesia.

On the other hand, Tocopheryl PEG 1000 succinate (TPGS), a water-soluble derivative of vitamin E, was explored as an enhancer for dermal penetration of drugs [124]. In particular, the TPGS-modified cationic NLC was studied to examine the skin permeation and analgesic effect of lidocaine. The results revealed that the TPGS-modified NLC was able to act as an appropriate delivery system for efficient and prolonged skin analgesia.

### Other nanotechniques for painless needles

The use of appropriate techniques and safe methods to avoid complications is the main goal of any injectable processes, and patient’s consent for these procedures is also very important. Since needles used for these processes are the main cause of pain, the selection of an ideal needle by physicians can significantly affect patient satisfaction levels. The influence of needle thickness on pain perception has been reported in various studies, in which a decrease in needle thickness could effectively reduce pain perception, thus increasing patient satisfaction [125–129].

For years, hypodermic needles and syringes have been applied for drug delivery. Recently, the emergence of micron-size needles provides more patient satisfaction, along with maximum transdermal drug delivery. Furthermore, nanoneedles are the new series of needles performed next to microneedles and expected to provide better penetration profiles. Microneedle includes arrays of micron-size needles. When used, they can deliver drugs to the dermal microcirculation without stimulation of any dermal nerves because of their short length of needles [130–132]. Nanoneedles and microneedles have the same principle of action, however, nanoneedles are small enough to be practically invisible. These features of nanoneedles can provide many advantages on painless operations [133].

**Fig. 6:**
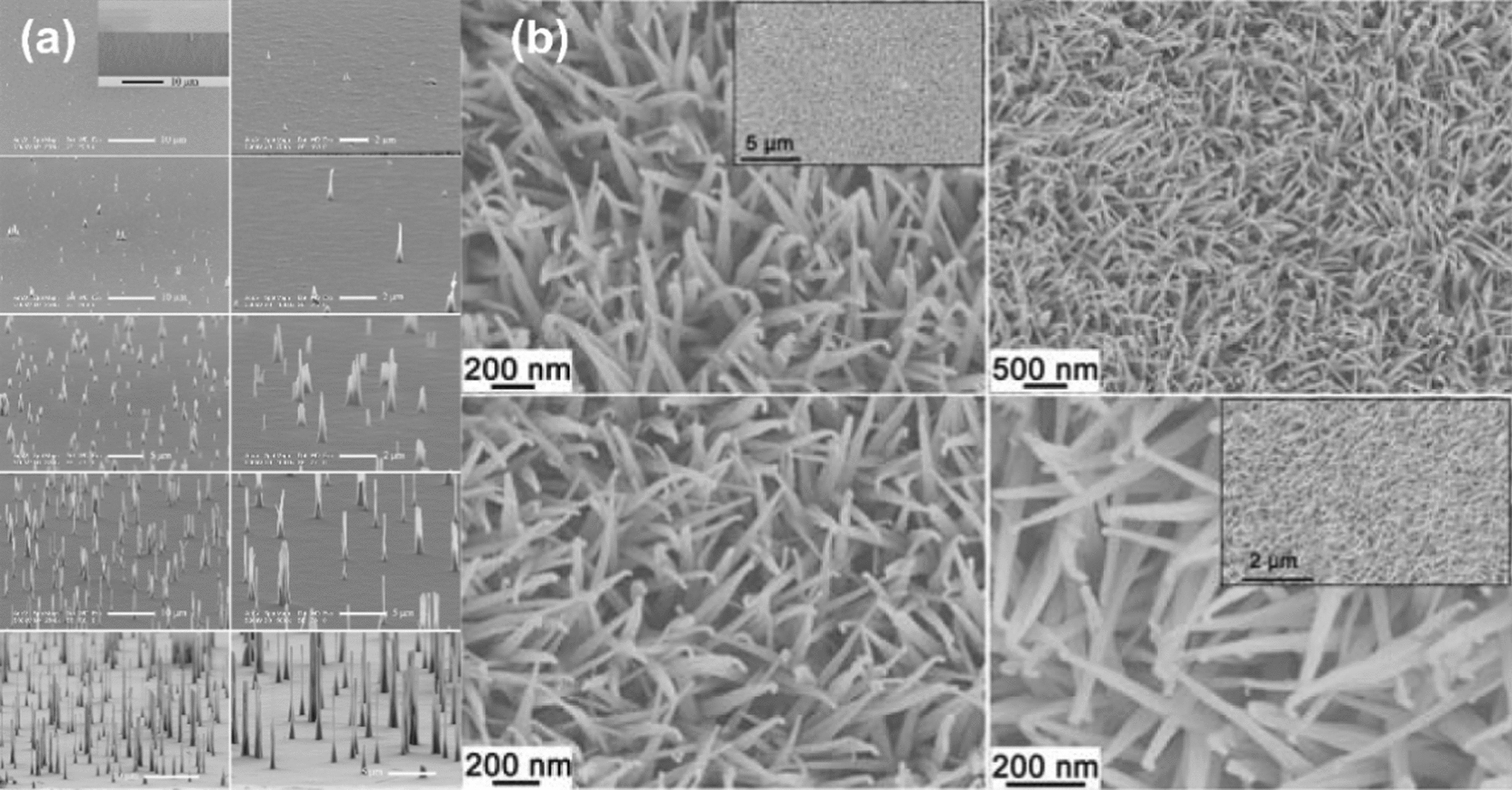
Scanning electron microscopy images of (**a**) diamond nanoneedles (reproduced with permission from Ref. [136], Royal Society of chemistry 2015). **b** Crystallized NiCo2O4 nanoneedles (reproduced with permission from Ref. [135], Royal Society of chemistry 2012)

In this regard, the nanoneedle technology has been recently focused in the field of nanomedicine. Nanoneedles can bypass cell membranes, and thus facilitate interactions with the intracellular milieu allowing drug delivery and intracellular sensing. For this purpose, Chiappini et al*.* developed a porous silicon nanoneedle system for the cytosolic delivery of CdTe quantum dots [134]. In addition to the silicon nanoneedle, NiCo_2_O_4_ nanoneedle [135] and diamond nanoneedles [136] are also examples of nanoneedles that have been introduced in recent years (Fig. 6). Still, further research and developments are needed to understand the full potential of nanoneedles and improve the accessibility of this nanotechnology to biological scientists. Challenges in case of nanoneedles are large quantities of production, and this emerging technology of nanoneedles has multidisciplinary nature, *i.e.*, its development requires collaboration of various fields. It is expected that nanoneedles will provide new opportunities to resolve many problems in biology.

### Dental procedures by nanoparticles

Few years ago, nanodentistry based on the use of nanomaterials was introduced as a science related to treatment, diagnosis, pain relief, and prevention of oral and dental diseases [137]. The use of nanotechniques in dentistry has changed attitude toward dentistry because it could provide the concept of pain-free and minimally-invasive dental procedures. Local anesthetics (LAs), including lidocaine, benzocaine, and tetracaine, are common anesthetic drugs used in dentistry [138]. Encapsulation of these drugs into liposome, cyclodextrins, lipid nanoparticles, hydrogels, and patches could not only prolong the analgesic effects, but also reduce the toxicity of local anesthetics [139]. In particular, the encapsulation of LAs into nanoliposomes was able to offer a suitable strategy for pain-free dental procedures.

Franz-Montan et al*.* developed the 5% lidocaine-loaded liposome and compared it with the liposome-encapsulated 2.5% eutectic mixture of lidocaine and prilocaine (EMLA) and 5% lidocaine ointment (*xylocaina*). The results revealed that the liposome-encapsulated 5% lidocaine and EMLA demonstrated the best in vivo topical anesthetic efficiency and better in vitro penetration parameters [140]. In another study by the same group, the effect of the liposome-encapsulated 2% ropivacaine was also examined in terms of topical anesthesia and pulpal response [141]. Several other formulations such as 20% benzocaine gel, placebo gel, and liposomal placebo gel were also compared with this formulation. The corresponding results showed that the liposome-encapsulated 2% ropivacaine and 20% benzocaine gel were most effective in reducing pain during needle insertion and soft tissue analgesia, however, they could not induce pulpal anesthesia after 15 min. In addition, SLNs [142], NLCs [143], and polymeric nanoparticles [144] are also other nanoparticle techniques that have been used with analgesic drugs to provide analgesia in dental procedures. Topical administration of these nanoparticles could effectively alleviate discomfort and pain perception in invasive events, such as needle insertion, during dental procedures. Dentin hypersensitivity is a sharp and short pain triggered by air, heat, cold, touch, and osmotic and chemical stimulants.

On the other hand, applying a thin coating on the exposed tooth can act as an occlusion and consequently block the pain stimulation. From last few years, fluoride has been known as an essential agent for dental remineralization. In recent, researchers have begun to focus on the efficiency of hydroxyapatite for remineralizing tooth enamel as the treatment of dental caries. From the viewpoint of remineralization properties, nanohydroxyapatite, similar to fluoride, is regarded as a satisfactorily promising agent against dentin hypersensitivity. Indeed, toothpastes comprising the nanohydroxyapatite could successfully decrease the duration of tooth sensitivity in patients who used tooth whitener without a desensitizing agent [145].

### Nanoparticles for neuropathic pain

Neuropathic pain is an intricate chronic pain disorder associated with the sense of stimulation or burning in a specific area; it is related to nerve injury and its unpleasant condition can affect the quality of life [146]. There are two general categories of neuropathic pain: first, the results from damage or disorders in peripheral nervous system, and second, the results from damage or disease in CNS. Pathology of neuropathic pain in the disorders in peripheral nervous system involves myelinated A-fibers and small unmyelinated C-fibers. Infectious diseases (*e.g.*, AIDS), diabetes mellitus, chemotherapy, and inflammatory and immune disorders are the most clinically diagnosed cases that create peripheral neuropathic pain [147, 148], whereas diseases or damages in the brain or spinal cord create central neuropathic pain.

Neuropathic pain is often caused by brain disorders such as neurodegenerative diseases (Parkinson’s disease) and cerebrovascular disease (post-stroke pain) [149]. Despite availability of numerous pharmaceutical drugs, the treatment of neuropathic pain is often ineffective [150]. To overcome these challenging conditions, a multidisciplinary approach has emerged for the treatment of neuropathic pain, in particular, nanomedicine with various capacities has shown great abilities to resolve these limitations [151, 152]. Various nanotechniques using nanomaterials could be effective in neuropathic pain management because of the unique features thereof that aid in diagnosis and therapeutic application at the cellular and molecular levels.

Relief of symptoms and palliative treatment in some pathological conditions are the main goals of neuropathic pain management [150]. In many patients, treatment of neuropathic pain is difficult and poorly effective. Opiate analgesics, NSAIDs, and antidepressants are not recommended for the treatment of neuropathic pain [153]. Drugs based on cannabinoid are emerging drugs that have been used to treat chronic pain, such as neuropathic pain. Psychological activity reduction as a side effect of cannabinoid drugs can help to improve therapeutic ratios. This improvement can be achieved by nanoparticle-based drug delivery systems. New synthetic cannabinoid receptor agonists, such as CB13, are relatively efficient in neuropathic pain treatment. The targeting of CB1 and CB2 receptors by CB13 could reverse mechanical hyperalgesia in neuropathic pain [154, 155].

Berrocoso et al*.* formulated an oral form of PEGylated PLGA nanoparticles containing CB13, and the corresponding neuropathic pain management by the formulations was evaluated by paw pressure and acetone tests in the neuropathic animal model [156]. Free CB13 and 3 types of the nanoparticle-encapsulated CB13 (*i.e.*, CB13-loaded PLGA nanoparticles, CB13-loaded PLGA + PEG nanoparticles, and CB13-loaded PLGA–PEG) were orally administrated at different doses. As a result, it was observed that free CB13 and the three types of nanoparticle cases exhibited the same analgesic effect, but the PEG-coated nanoparticles exhibited a very prominent analgesic ability with the longest and continued pain reduction effect.

As mentioned above, one of the major limitations of cannabinoid receptor agonists is psychoactivity. Micelles, which are sphere-shaped aggregates of amphiphilic molecules, can effectively reduce the effect on CNS by restricting transport across the blood–brain barrier (BBB), furthermore, nanomicelles can prolong plasma half-life by escaping renal elimination because of their submicron size. These can be the reasons for the effectiveness of nanomicelles in the treatment of neuropathic pain by cannabinoids. Cannabinoids such as WIN55,212-2 (WIN) and CP55,940 are strong CB1 and CB2 receptor agonists, respectively. Linsell et al*.* encapsulated WIN in the styrene maleic acid micelles (SMA), and then evaluated neuropathic pain management using the chronic constriction injury (CCI) model of sciatic neuropathy [157]. Intravenous injection of the water-soluble SMA-WIN micelles with the diameter of 6 nm could retain their formulations in the peripheral nervous system and could produce prolonged analgesia in comparison with the base WIN.

After nociceptor sensitization, the inflammatory mediators, signaling molecules, and immune cells infiltrated in the inflamed nerve, triggering pain perception. This inflammatory phenomenon that is largely produced by the infiltration of macrophages leads to the expression of cyclooxygenase-2 enzyme, which is responsible for the production of PGE2 [158–161]. NSAIDs are a group of drugs that can reduce the pain resulted from the nerve damages associated with the inflammation by inhibiting prostaglandin production. The macrophages can not only promote, but also prevent inflammation. They can switch phenotype to exhibit these actions by obtaining either proinflammatory (termed M1) or anti-inflammatory (termed M2) phenotypes [162–165]. To relieve neuropathic pain associated with macrophage polarity, Saleem et al*.* developed theranostic approach containing both therapeutic (anti-inflammatory drug celecoxib) and diagnostic (near-infrared fluorescent dye) nanoemulsions to distinguish the anti-inflammatory and proinflammatory phenotypes of infiltrating macrophages at the site of sciatic nerve injury. Their results showed that the cause of chronic pain relief was an alteration in M2 macrophage phenotype; this type of macrophages fuse to form MGCs at injured sciatic nerve. Thus, at the injured nerve, the change to an anti-inflammatory environment led to a decrease in M1 macrophage employment, reducing inflammation and neuropathic pain perception [166].

**Fig. 7:**
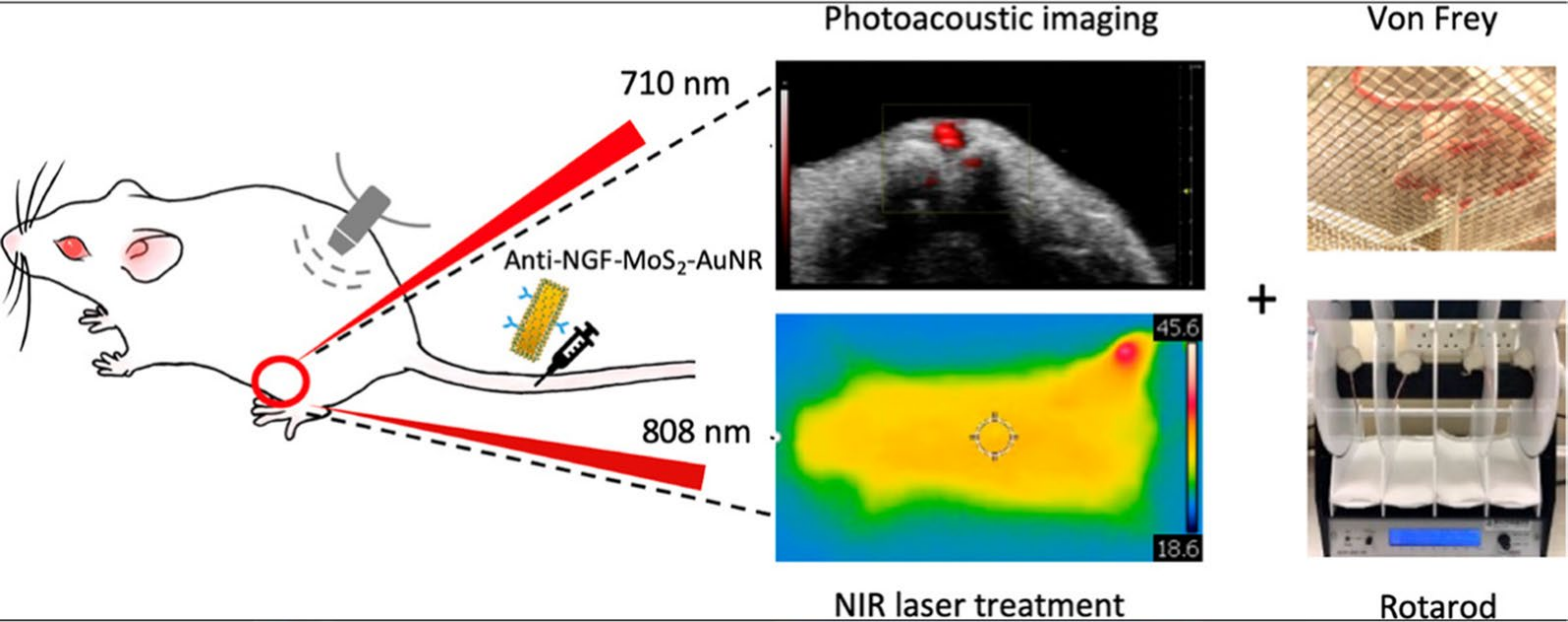
Schematic representation of theranostic approach to reduce osteoarthritis pain in rats by conjugation of NGF antibody with molybdenum disulfide nanosheet-coated gold nanorods (MoS2-AuNR) for photoacoustic imaging and photothermal analgesic treatment. Von Frey and rotarod tests were performed to evaluate the locomotive ability and balance of the animals, respectively. (reproduced with permission from Ref. [172], American Chemical Society 2021)

Recently, satisfactory results also have been reported for management of osteoarthritis pain based on the theranostic approach. Osteoarthritis is a common chronic inflammatory disease and a major cause of joint pain, its progression can be manifested by loss of function and finally permanent disability. To date, no medication is available for the absolute treatment of osteoarthritis, and most therapies are palliative in nature and reduce symptoms. This is probably due to the inadequate understanding of the early signs and methods of diagnosis, which has turned this disease into a global problem [167]. Resolving this issue requires the development of imaging-based assessment tools to accurately and quantitatively determine the origin of peripheral osteoarthritis pain, which will actually lead to a change in current analgesics and surgical use. In response to mechanical or inflammatory stimuli, the nerve growth factor (NGF), which plays a key role in activating and sensitizing peripheral nerves, is secreted by articular chondrocytes and synovial fibroblasts, triggering pain in an osteoarthritis joint [168, 169]. In a mouse model of post-traumatic osteoarthritis induced by destabilization of medial meniscus (DMM), the level of synovial NGF protein exhibited a significant increase at 4 weeks after surgery [170]. Therefore, NGF-targeted molecular imaging will assist to accurately visualize the source of osteoarthritis pain. Recently, functionalized gold nanoparticles have been considered as a common choice for theranostic agent due to their excellent optical properties [171]. Conjugation of NGF antibody with molybdenum disulfide nanosheet-coated gold nanorods (MoS2-AuNR) nanoprobes has been shown to active targeting of NGF on painful knees in an osteoarthritis surgical murine model (Fig. 7) [172]. Wherein, addition of MoS2 coating significantly improved the photoacoustic and photothermal performance of AuNR. In this study, it was revealed that the presence of functionalized nanoprobes in the osteoarthritis knee was higher than the intact knee, and intensity of mechanical allodynia was related to the accumulation amount in the mouse model. Therefore, this study provided conditions that under the guidance of imaging, NIR-stimulated photothermal therapy could reduce walking imbalance behavior and mechanical allodynia for subacute and chronic stages of osteoarthritis. As a result, this theranostic approach will provide a new window for accurately identification of the pain and simultaneously and effectively pain management in the future.

Development of chronic pain in the CNS is significantly dependent on the glial cells, *i.e.*, microglia and astrocytes, and in the physiological conditions both microglia and astrocytes are not in a proliferating state [173–175]. Recent studies demonstrated the effect of mitogen-activated protein kinases (MAPKs) in the development of neuropathic pain in the CNS. Moreover, after external damage leading to a pathological condition, some studies reported that a Ras family member (Rheb) was upregulated in the mTOR pathway [176, 177]. Zoledronic acid is a type of pharmacological agent used for the treatment of bone demineralization by inhibiting isoprenylation of Ras family proteins. This could help in the neuropathic pain treatment by inhibiting the MAPK cascade. Unfortunately, the pharmacokinetic profile of zoledronic acid has been one of the biggest limitations [178]. To increase the bioavailability of zoledronic acid and reduce its bone binding, zoledronic acid was encapsulated in the liposome-based formulation (LipoZOL). Because of partially or disrupted BBB in chronic neuropathic pain, the LipoZOL could pass through it. Consequently, the accumulation of LipoZOL in the CNS and presence of proinflammatory cytokines were assessed in an animal model of neuropathic pain [177]. In the activated microglia, the phosphorylation of p38 MAPK has been detected, which led to the production of proinflammatory mediators, resulting in amplified pain symptoms. Therefore, reduction or inhibition of p38 MAPK phosphorylation has the potential of reducing neurologic pain [179, 180].

**Fig. 8:**
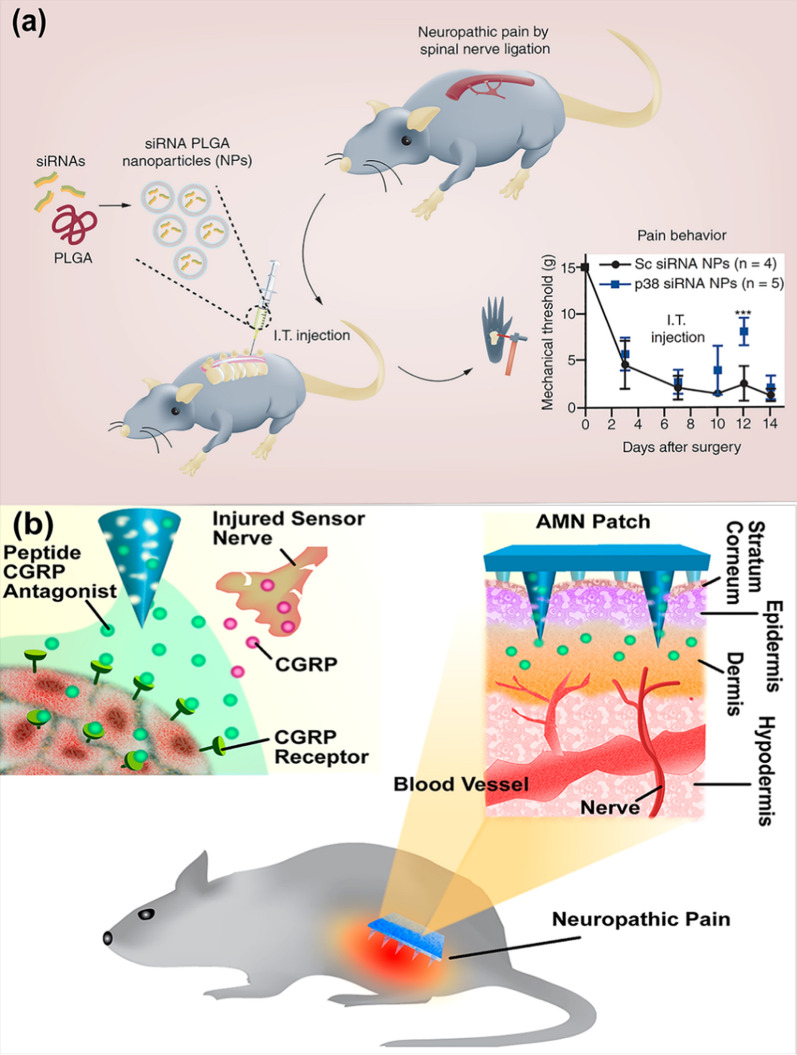
**a** Scheme of study that evaluated the effect of p38 siRNA PLGA nanoparticles on neuropathic pain (reproduced with permission from Ref. [181], Future Medicine 2018). **b** Schematic of dissolvable MNs mediating local delivery of peptide CGRP antagonist to produce analgesia for the treatment of neuropathic pain (reproduced with permission from Ref. [187], American Chemical Society 2017). *siRNA* small interfering RNA, *PLGA* poly(lactic-co-glycolic acid), *I.T. injection* intrathecal injection, *CGRP* calcitonin gene-related peptide

In recent, there is noticeable attention to the gene therapies based on small interfering RNA (siRNA) silencing of genes-related disease because of its potential to treat genetic disorders. For siRNA delivery, Shin et al*.* selected PLGA nanoparticles because its safety has already been established by the FDA and it has been displayed to be simply endocytosed by microglial cells (Fig. 8a) [181]. In another in vivo study, gold nanoparticles were selected as carriers for siRNA delivery [182]. In response to inflammation or trauma, a calcitonin gene-related peptide (CGRP) synthesized and released by nociceptive sensory neurons has the main effect in the maintenance and development of neuropathic pain. Systemic blocking of CGRP receptors has shown potential in the treatment of migraine [183–186]. An analgesic microneedle (AMN) patch with a CGRP antagonist peptide was transdermally administered for the local neuropathic pain treatment. Local analgesic effects were assessed in rats and it was observed that the use of microneedle patches containing CGRP antagonist peptide was a safe and simple approach for the treatment of neuropathic pain (Fig. 8b) [187].

### Current clinical studies

Not only is pain a very complex ubiquitous phenomenon, but the experience of pain is also significantly different in each individual. On the other hand, the current information in understanding the subjective nature of pain and the variety in the pain assessment methodologies have significantly improved the quality of clinical trials for prospective pain therapies [188]. In the mid-1950s, clinical trials of pain treatment launched with the innovative work of Henry Beecher and Louis Lasagna, who directed randomized two-group trials on main nonsteroidal anti-inflammatory drugs and opioid analgesics [189]. Henry Beecher also explored the complications of accurate measurement of pain, the limitations in finding of specific patient’s population, the extent and prevalence of placebo effects, and the lack of a complete definition of response to treatment [190].

**Fig. 9:**
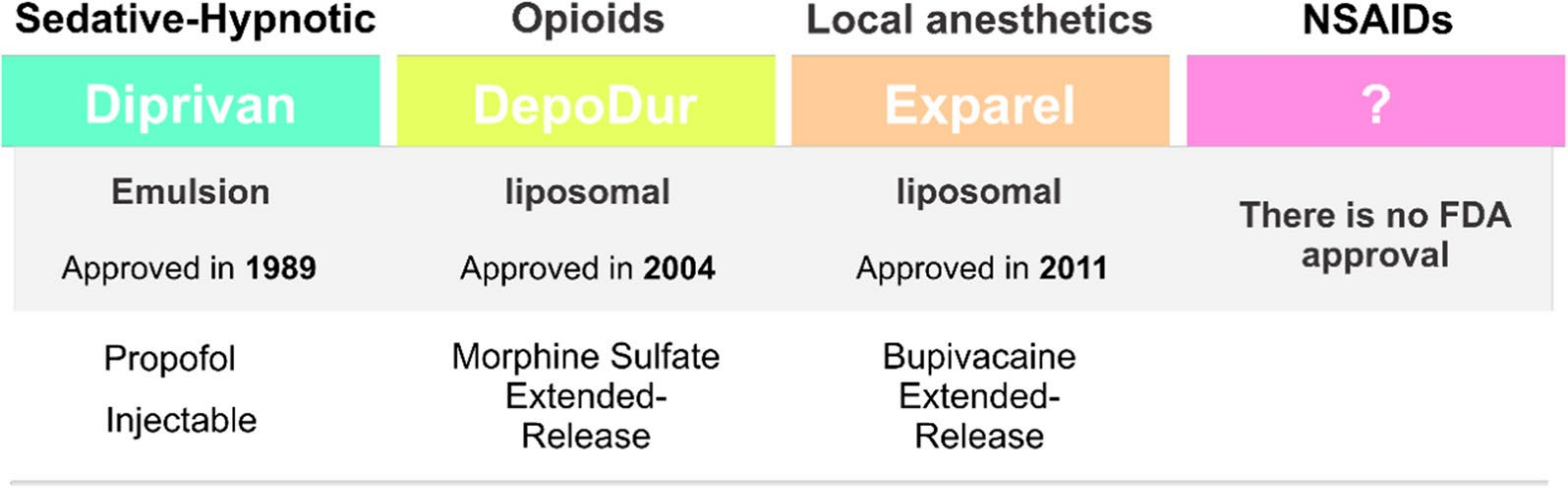
FDA approved nanodrugs for the pain management

However, in spite of such clinical trials, many of these challenges still exist in recent pain studies. Accordingly, many clinical trials are underway to treat various types of pain, including pain associated with cancer, headaches, arthritis, neurological problems, and abdominal pain. In recent decades, nanomedicine has achieved conisderable progress in clinical use with the use of numerous micro and nanodrugs. Nevetheless, few clinical trials with using nanoparticles have been conducted and limited cases have been approved by the FDA (Fig. 9). For example, Diprivan®, which is an injectable emulsion of propofol, was as the first nanodrug in its class approved in 1989 by FDA for its application in the initiation and maintenance of general anesthesia or as a sedative in adults [191]. Subsequently, various modifications have been made to the emulsion formulations to overcome the disadvantages of propofol emulsions such as the pain on injection and the risk of infection [192–196]. In addition, a number of propofol formulations with novel nanocarriers have been studied and evaluated in preclinical studies [197].

Liposomes are among the first nanodrug carriers to be successfully translated into clinical applications. Hence many liposomal analgesic formulations have been developed and are currently on the market. In particular, administration of bupivacaine loaded liposomes has been shown to be extremely effective with acceptable pharmacodynamics and pharmacokinetics properties and lower systemic toxicity [198, 199]. Injectable liposomal bupivacaine, commercially named Exparel™, is the only extended-release liposomal local anesthetic which is approved by FDA in 2011 for administration into the surgical site to create postsurgical analgesia in adults [191]. Although it was initially developed for postsurgical analgesia and is still used for this purpose [200–204], its scope has expanded and it has been used successfully in epidural injections [205].

Opioids are established as the “gold standard” in clinical practice for management of postoperative pain. Epidural morphine sulfate provides excellent and prolonged postoperative analgesia compared to systemic administration. DepoDur^®^ that is a novel liposomal encapsulated extended-release epidural morphine was also approved by FDA for the pain relieve following major surgery [191]. The effectiveness of DepoDur^®^ has been reported in a variety of clinical trials executed in patients undergoing major surgeries, such as colon resection [206], hip arthroplasty [207] and cesarean section [208]. The therapeutic efficacy of DepoDur^®^ was also assessed in patients (n = 280) undergoing open and laparoscopic colorectal procedures, where was used between July 2010 and April 2012 [206]. Primary outcome, including resting and dynamic pain, mobilization, and need for additional analgesia, were evaluated. Sufficient pain management was realized at 24 and 48 h, wherein 81% of the patients required simple analgesia in 24 h and 62% of themrequired in 48 h. 79% of the patients were mobilized in 24 h and 88% of them were able to move in 48 h. Overall, this study showed that DepoDur^®^ is an effective alternative to common pain management techniques. NSAIDs are the most widely prescribed drugs for acute and chronic pain due to their analgesic, anti-inflammatory, antipyretic properties, and their no dependence or addiction potential. However, it is known that these agents are associated with significant gastrointestinal and cardiovascular adverse events. In an attempt to enhance the efficacy and reduce the toxicity of NSAIDs, nanocarriers have been used in many preclinical studies to obtain targeted delivery to the site of inflammatory pain. However, the number of NSAID nano-formulations conducted in the clinical trials is very limited, and none have been approved by FDA to date [21].

### Future perspective

Strong potential of nanotechnology offers exceptional opportunity to address many of unsolved problems in pain management. Accompanied by these abilities, multidisciplinary team works also are necessary to achieve ultimate advances in this field. Therefore, simultaneously efforts should be done toward growth of researches about effects of nanoparticles on different pain pathways, development of pain biomarkers detection techniques, expansion of tissue engineering researches to minimize pain perception associated with nerve injuries, and introducing of up-to-date and advanced sciences, such as gene therapy and CRISPR in the field of pain management by targeting specific diseases or genes that cause pain, as well as the development of appropriate and safe vectors to deliver genetic material to the source of pain. Finally, commercialization of appreciated studies and their impact on clinical treatments require successful integration between scientific and governmental centers.

## Conclusions

Sufficient pain management remains one of the major medical challenges. Pharmacological and nonpharmacological approaches are used for the treatment of mild to severe pain. However, these treatments have still not provided sufficient patient satisfaction, particularly in chronic pain. pain management with employing nanotechnology has recently emerged as a new approach to improve the quality of treatment. Nanotechnology has created a new perspective for pain treatment by introducing of nanomaterials with analgesic properties to satisfactorily pain modulation, engineering of multifunctional nanocarriers to improve the low therapeutic index of conventional analgesic drugs, accurate detection of pain biomarkers along with use of appropriate techniques to avoid complications is the pain mangement. Therefore, successful combination of these factors is expected to achieve ultimate pain control without side effects. Despite many advances of nanotechnology in the preclinical studies, clinical trials on the further development of these systems are still limited. Therefore, efforts should be directed to further develop these nanosystems. This requires interdisciplinary teamwork including researchers, physicians, and engineers in addition to provision of government and academic support.

## Data Availability

Not applicable.

## Abbreviations

PLGA: Poly (lactic-co-glycolic acid)
ZnO: Zinc oxide
NMDA: N-methyl-d-aspartate
GABA: γ-Aminobutyric acid
MgO: Magnesium oxide
MnO2: Manganese dioxide
CNS: Central nervous system
Fe3O4: Magnetite
MRI: Magnetic resonance imaging
ROS: Reactive oxygen species
Ropiv: Ropivacaine
NSAIDs: Nonsteroidal anti-inflammatory drugs
FDA: Food and drug administration
GI: Gastrointestinal
PEG: Polyethylene glycol
RGD: Arg–Gly–Asp
SLNs: Solid lipid nanoparticles
NLCs: Nanostructured lipid carriers
PWTP: Paw withdrawal threshold to pressure
PLA: Polylactic acid
PCL: Polycaprolactone
EMLA: Eutectic mixture of local anesthetics
EPO: PH sensitive cationic polymer consisting of 1:2:1 ratio of methyl methacrylate, N_N-dimethylaminoethyl methacrylate, and butyl methacrylate monomers
IL-1β: Interleukin‐1β
TNF-α: Tumor necrosis factor α
DMAB: Didodecyldimethylammonium bromide
LPNs: Lipid–polymer hybrid nanoparticles
TPGS: Tocopheryl PEG succinate
CdTe: Cadmium telluride
NiCo2O4: Nickel cobaltite
LAs: Local anesthetics
AIDS: Acquired immunodeficiency syndrome
BBB: Blood–brain barrier
CB: Cannabinoid
SMA: Styrene maleic acid
MGCs: Multinucleated giant cells
MAPKs: Mitogen-activated protein kinases
Rheb: Ras family member
mTOR: Mammalian target of rapamycin
LipoZOL: Zoledronic acid encapsulated into liposomes
siRNA: Small interfering RNA
CGRP: Calcitonin gene-related peptide
AMN: Analgesic microneedle
MoS2-AuNR: Molybdenum disulfide nanosheet-coated gold nanorods
DMM: Destabilization of medial meniscus
NGF: Nerve growth factor
cAMP: Cyclic adenosine monophosphate
TENS: Transcutaneous electrical nerve stimulation
COX: Cyclooxygenase
